# Effects of posterior tibial slope on the mid-term results of medial unicompartmental knee arthroplasty

**DOI:** 10.1186/s42836-021-00070-y

**Published:** 2021-04-12

**Authors:** Zhijie Chen, Kaizhe Chen, Yufei Yan, Jianmin Feng, Yi Wang, Zhihong Liu, Qingming Yang, Chuan He

**Affiliations:** Department of Orthopaedics, Shanghai Key Laboratory for Prevention and Treatment of Bone and Joint Diseases, Shanghai Institute of Traumatology and Orthopaedics, Ruijin Hospital, Shanghai Jiao Tong University School of Medicine, 197 Ruijin 2nd Road, Shanghai, 200025 People’s Republic of China

**Keywords:** Unicompartmental knee arthroplasty, Tibial plateau osteotomy, Posterior tibia slope, Maximal knee flexion

## Abstract

**Objective:**

To evaluate the effect of medial posterior tibial slope (PTS) on mid-term postoperative range of motion (ROM) and functional improvement of the knee after medial unicompartmental knee arthroplasty (UKA).

**Methods:**

Medical records of 113 patients who had undergone 124 medial UKAs between April 2009 through April 2014 were reviewed retrospectively. The mean follow-up lasted 7.6 years (range, 6.2–11.2 years). Collected were demographic data, including gender, age, height, weight of the patients. Anteroposterior (AP) and lateral knee radiographs of the operated knees were available in all patients. The knee function was evaluated during office follow-up or hospital stay. Meanwhile, postoperative PTS, ROM, maximal knee flexion and Hospital for Special Surgery (HSS) knee score (pre−/postoperative) of the operated side were measured and assessed. According to the size of the PTS, patients were divided into 3 groups: group 1 (<4°), group 2 (4° ~ 7°) and group 3 (>7°). The association between PTS and the knee function was investigated.

**Results:**

In our cohort, the average PTS was 2.7° ± 0.6° in group 1, 5.6° ± 0.9° in group 2 and 8.7° ± 1.2° in group 3. Pairwise comparisons showed significant differences among them (*p* < 0.01). The average maximal flexion range of postoperative knees in each group was 112.4° ± 5.6°, 116.4° ± 7.2°, and 117.5° ± 6.1°, respectively, with significant difference found between group 1 and group 2 (*p* < 0.05), and between group 1 and group 3 (*p* < 0.05). However, the gender, age, and body mass index (BMI) did not differ between three groups and there was no significant difference between groups in terms of pre−/postoperative HSS scores or postoperative knee ROM.

**Conclusion:**

A mid-term follow-up showed that an appropriate PTS (4° ~ 7°) can help improve the postoperative flexion of knee. On the other hand, too small a PTS could lead to limited postoperative knee flexion. Therefore, the PTS less than 4° should be avoided during medial UKA.

## Introduction

UKA has been increasingly used in clinical practice over the last decade [1]. For the treatment of end-stage medial compartment disease of the knee, it has the advantage of minimal invasiveness, less bleeding, less soft tissue injury, fast recovery and bone-stock conservation [2, 3]. It has become a popular alternative to total knee arthroplasty (TKA), with great patient satisfaction and favorable functional outcomes, especially in younger patients [4]. In recent years, medical centers that performed a large number of UKAs reported that the 10-year prosthesis survival rate after UKA stood somewhere between 94% and 98% [5–7], which is close or even equal to the post-TKA rate. Despite the success, complications in UKA were reported, and complications, such as prosthesis loosening and lining wear, which are associated with mal-alignment after UKA, accounted for over 50% of the reasons for UKA revision [8].

It is more technically demanding to align and seat UKA prostheses appropriately given small operation space associated with minimally invasive procedures. Although Berger et al. [5] followed up 62 patients and found a 92% postoperative satisfaction, postoperative radiographic examination still revealed progressive loss of joint space. The statistics of the Australian Orthopaedic Association National Joint Replacement Registry [9] showed that the cumulative percentage of 5-years UKA revision was as high as 15%. The revision of UKA is associated with multiple factors, including prosthesis design, surgeon experience, patient selection, polyethylene quality, and intraoperative alignment and fixation [10–13]. Among them, the accurate positioning of the implant is a critical factor for implant longevity. A broad range of up to 20° for the PTS has been recommended by many researchers and manufacturers [14], and the PTS may directly affect implant stability, postoperative joint mobility, and the wear rate of the prosthesis. Takayama et al. [15] showed that, compared with knee flexion, an increased PTS led to a tight component gap at knee extension. Therefore, they suggested that when tibial sagittal osteotomy is performed, anatomical PTS should be individualized. However, no unified standard is available for the optimal PTS, as the role of PTS is not fully understood and remains controversial.

In this study, we retrospectively investigated the effect of PTS on the knee function in the medium term after medial UKA, with an attempt to find a surgical strategy for the tibial plateau osteotomy in the medial UKA.

## Materials and methods

### Patients

We retrospectively analyzed the records of 113 patients with medial unicompartmental knee osteoarthritis who had undergone medial UKA between April 2009 and April 2014. There were 38 males (involving 43 knees) and 75 females (involving 81 knees), with an average age of 68.3 years (range, 48 to 86 years). Mean postoperative follow-up lasted 7.6 years (range, 6.2–11.2 years). The inclusion criteria [16] for this case series study included: (1) patients who were definitely diagnosed with medial unicompartmental knee osteoarthritis, (2) preoperative ROM > 90°, (3) joint flexion contracture < 15° and (4) varus deformity < 15°. The exclusion criterion was the past history of having undergone knee surgeries. The anteromedial osteoarthritis of knee with normally functional anterior cruciate ligament is considered to be best indicated for UKA [6]. It is worth noting that patellofemoral joint osteoarthritis was not a contraindication for UKA in this study [17]. Careful and reasonable patient selection was the key to the efficacy of the surgery.

### Follow-up

**Fig. 1:**
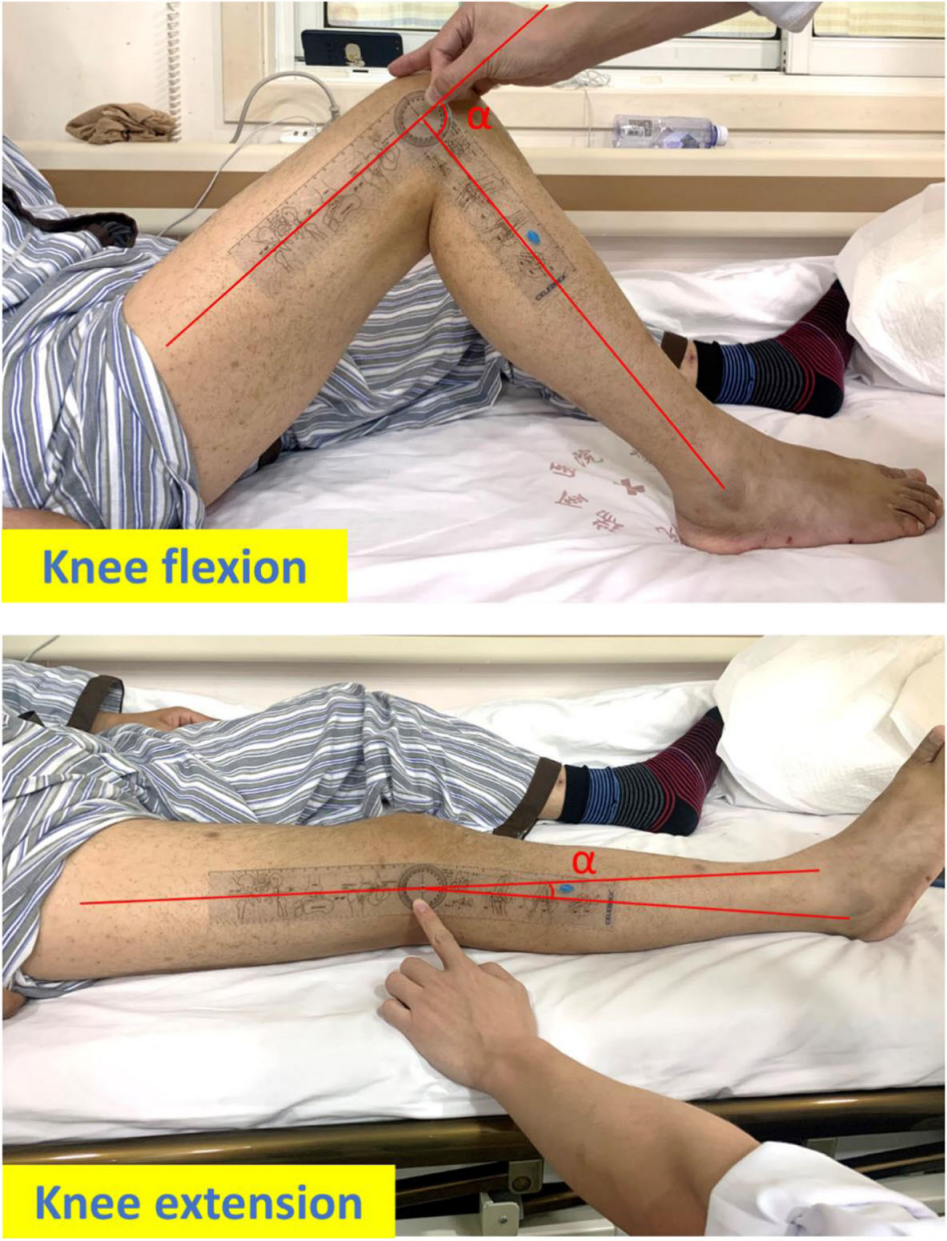
The measurement method of extension angle/flexion angle defined by α-angle

The patients were followed up for HHS scores, and AP and lateral radiographic results (DICOM format) of the knee. In the physical examination, we employed a protractor to measure ROM and maximal flexion range of the knees (Fig. 1).

### Surgical technique

All surgical procedures were performed by a senior surgeon (HC) using the same prosthetic implant (Bioment, Oxford III, USA) by using the same minimally invasive procedure, including the tibial plateau osteotomy. Following the operation manual of Biomet Oxford phase III, the prosthesis was aimed for a 7° of PTS.

### Radiological analysis

**Fig. 2:**
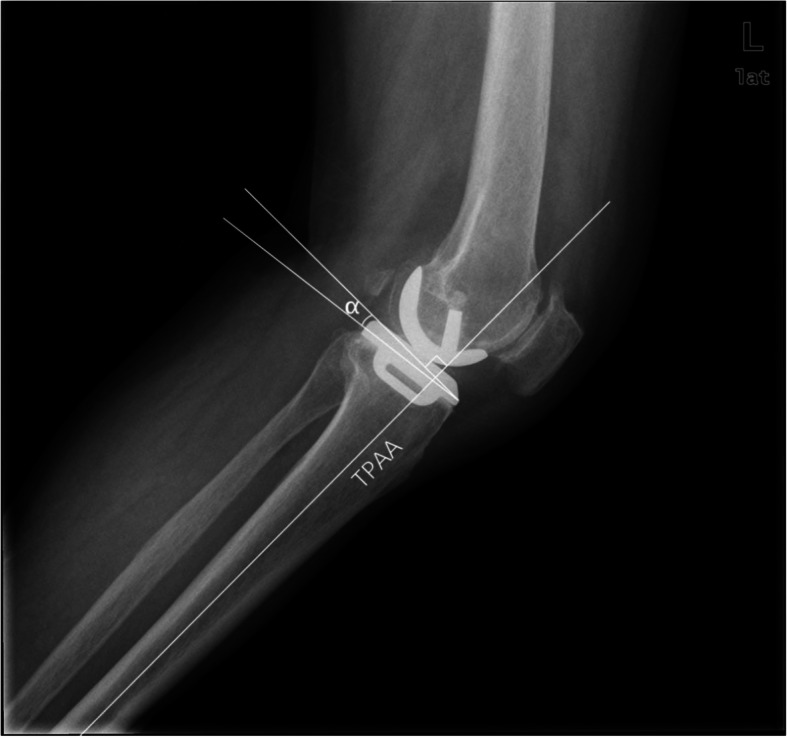
The measurement method on standardized knee radiographs in the lateral view. TPAA, the tibial proximal anatomical axis. Tibial posterior slope (PTS) was defined by the angle between the posterior inclination line of the medial tibial plateau and a line perpendicular to the TPAA, which is defined by α-angle

Postoperative lateral radiographs of the knee were obtained in DICOM format, and the PTS was measured by using the UniSight software and the measuring tool that goes with the package. The long axis of the proximal tibia, which was most commonly used in clinical practice and is believed to be the normal anatomical axis of the tibia [18], was taken as the reference axis. PTS was defined by the angle between the line perpendicular to the reference axis and the line parallel to the prosthesis platform (Fig. 2). Radiological parameters were independently measured by two authors (CZJ and CKZ) and checked by a senior doctor (HC) to ensure that PTS error was less than 1°. In terms of the angle of PTS measured, patients were divided into 3 groups: a <4° group (group 1), a 4° ~ 7° group (group 2), a >7° group (group 3).

### Statistical analysis

All statistical analyses were conducted by using SPSS version 19.0. Categorical variables were compared by using the Chi-square test. Continuous variables were presented as mean ± standard deviation (SD) and the ANOVA was applied to analyze statistical differences of the related data. The *t*-test and Student-Newman-Keuls test were used to evaluate the significance of differences between independent samples. *p* values in this study were two-sided and considered statistically significant when they were less than 0.05.

## Results

**Fig. 3:**
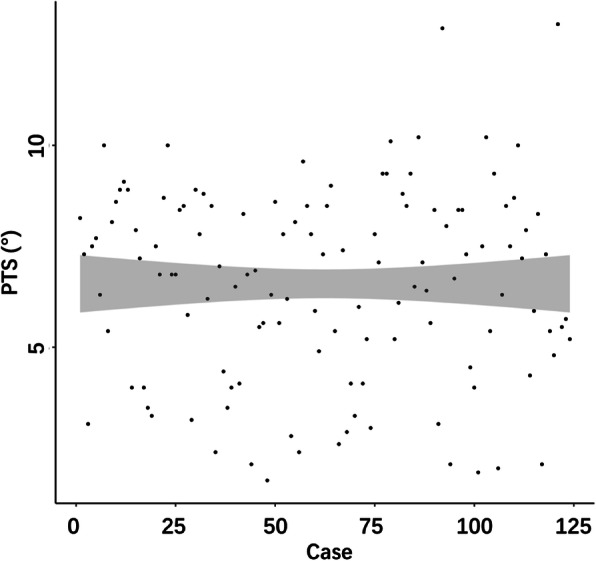
The scatter plot graphs illustrating the PTS distribution trend of 113 patients (involving 124 knees)

Of 124 knees included in the study, 19 knees (15.3%) were in group 1, 45 knees (36.3%) in group 2, and 60 knees (48.4%) in group 3. The average PTS of all knees was 6.6° ± 2.4°, ranging from 1.7° to 13°, with P_75_-P_25_ = 8.4°-5.0° (Fig. 3). And there were not significant differences among the three groups in gender, age, and BMI (Table 1).

**Table 1:** The baseline characteristics of the groups

	Group 1	Group 2	Group 3	*p* value
Male/Total	5/19	18/45	19/60	0.511
Average age (y)	68.16 ± 8.78	68.93 ± 7.04	67.83 ± 7.27	0.753
BMI (kg·m^−2^)	26.92 ± 3.26	26.06 ± 3.49	25.85 ± 3.47	0.500

Data are shown as mean ± standard deviation or numbers

Abbreviations: BMI body mass index

In our cohort, apart from aforementioned patients, 6 patients died and lost to follow-up, and 15 lost contact. No known complications of the UKA took place at the time of death in these patients. During the follow-up period, the insert replacement was performed on one patient due to the impingement of residuary cement and 4 patients received revision surgery with TKA. Of these patients, the PTS was 3.4°, 9.9°, 8.8°, 12.9° and 3.9°, respectively (Table 3). The remaining patients received no subsequent surgery or were not re-admitted due to UKA.

The postoperative HHS scores averaged 91.7 at the last follow-up, being virtually 40 higher than preoperative HSS scores, indicating that the clinical effect was excellent. Furthermore, the average PTS was 2.7° ± 0.6° in group 1, 5.6° ± 0.9° in group 2 and 8.7° ± 1.2° in group 3, respectively. Correspondingly, preoperative HSS score in each group was 48.79 ± 6.02, 52.40 ± 6.77, and 50.93 ± 7.02, respectively. And postoperative HSS score was 92.68 ± 3.23, 91.87 ± 4.15, and 91.67 ± 5.54 in groups 1, 2 3, respectively. Significant improvement was achieved after UKA (*p* < 0.01). However, there were no significant differences among the three groups in pre- and postoperative HSS scores. Similarly, as shown in Table 2, there was no significant difference in the ROM of both pre- and postoperative knee joints among the three groups (all *p* > 0.05). We are led to draw the conclusion that the PTS exerts little effect on the postoperative functional recovery after the medial UKA within a certain range.

**Table 2:** The perioperative knee function in the three groups

	Group 1	Group 2	Group 3	*p* value
Preoperative				
HSS scores	48.79 ± 6.02	52.40 ± 6.77	50.93 ± 7.02	0.148
ROM	99.70 ± 5.52	100.09 ± 5.11	99.37 ± 6.80	0.857
Maximal knee flexion	100.23 ± 5.31	99.95 ± 5.69	100.18 ± 5.99	0.973
Postoperative				
HSS scores	92.68 ± 3.23	91.87 ± 4.15	91.67 ± 5.54	0.720
ROM	115.79 ± 8.04	116.44 ± 7.20	117.50 ± 6.14	0.563
Maximal knee flexion	112.37 ± 5.62	116.44 ± 7.20	117.50 ± 6.14	0.013

Data are shown as mean ± standard deviation or numbers

Abbreviations: ROM: range of motion, HSS: Hospital for Special Surgery

**Fig. 4:**
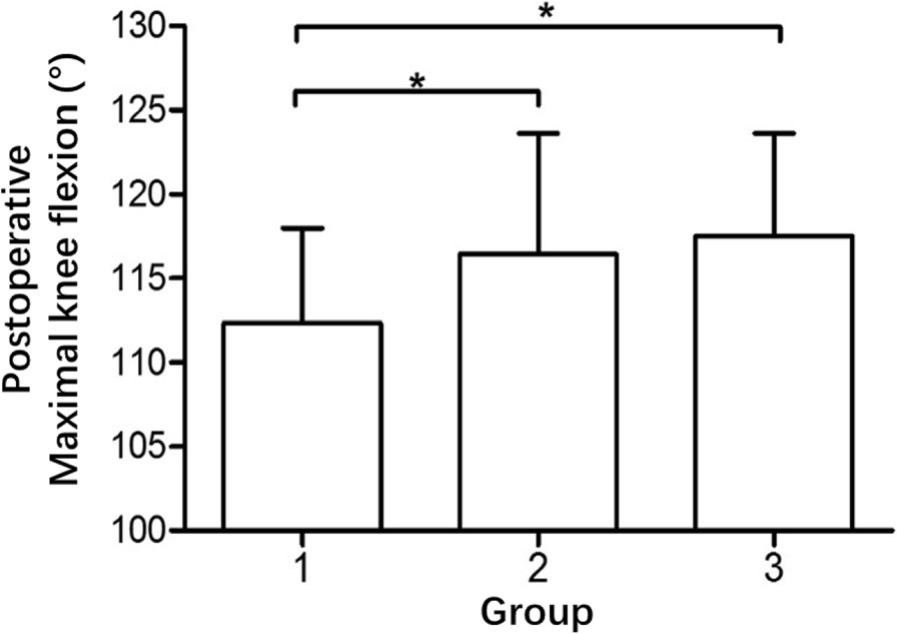
The histogram comparing the postoperative maximal knee flexion between the three groups with different ranges of PTS. *p < 0.05

Our main finding was that the value of postoperative maximal knee flexion was significantly lower in group 1 than in group 2 and group 3 (*p* = 0.013). It was 116.44° ± 7.20° in group 2 and 117.50° ± 6.14° in group 3 respectively, whereas it was 112.37° ± 5.62° in group 1, with the difference in flexion being almost 5° (Fig. 4). This finding indicated that the PTS has an impact on the maximal knee flexion after medial UKA. In performing tibial sagittal osteotomy during a medial UKA, too small a PTS slope (< 4°) may impair postoperative knee flexion.

## Discussion

Multiple studies examined the impact of PTS on the knee joint from different perspectives. Kang et al. [19] explored biomechanical effects of PTS on UKA using finite element analysis, and found that the PTS influenced the contact stress on the polyethylene insert and articular cartilage after UKA. Hashemi et al. [20] used MRI to assess PTS and Nunley et al. [21] employed CT scans to evaluate the PTS in patients who are indicated for UKA. Of note, both of them noted a significant difference between the medial and lateral compartments in tibial slopes. Medial compartment diseases tend to be associated with increased anterior wear and a decreased PTS slope, while lateral compartment diseases commonly bear connection with posterior wear and an increased PTS. Although the role of the PTS has been discussed in the current literature on UKA, its role and recommended angle remain elusive [15].

Excellent lower limb alignment and precise implant positioning are essential to the attainment of favorable results of UKA. Therefore, researchers have tried different measuring methods to find the optimal PTS. For example, Hashemi et al. [20] used the long axis of the proximal tibia as the reference axis to blindly measure the PTS of 20 cases. The PTS of male adults was − 3° ~ 10°, while the PTS of female adults was 0 ° ~ 10 °. They found a larger PTS in females (5.9 °) than in males (3.7 °). On the other hand, Zhang et al. [22] applied different reference axes to evaluate the PTS of 80 healthy adults in southern China on three-dimensional reconstruction of CT. The medial tibial posterior slope was 11.5° with the proximal tibia long axis, 8.4° with the anterior tibial cortical axis and 6.3° with the posterior tibial cortical axis. The results of the three axes varied but were correlated with each other significantly. In this study, according to the original design objective and the operation manual, the prosthesis (Oxford phase III, Biomet) was aimed for a 7° of PTS, a radiological standard. Although the surgeon strictly followed the steps as instructed, the PTS measured after medial UKA was 6.6° ± 2.4°, not a constant angle of 7° (Fig. 2). Various factors can impact on PTS measurements. First and foremost, when an extracorporeal tibial medullary locator is used, anatomical variations may lead to disparate positioning, which can eventually cause discrepancy in tibial plateau osteotomy. Secondly, patients with medial compartment osteoarthritis may well have subchondral osteosclerosis of the tibial plateau, which can result in swing saw blade deviating upward and a smaller PTS in osteotomy. Or, in order to avoid deviation, the surgeon may slightly press the swing saw downwards, which leads to excessive osteotomy of the tibial plateau. Last but not least, due to the use of minimally invasive incision in UKA, poor vision in the posterior part of the knee joint may cause excessive bone cement to remain, which elevates the posterior part of the tibial prosthesis and leads to a smaller PTS. What is more, factors such as varus deformity, valgus deformity and flexion contracture, need to be considered during the operation to avoid excessive correction of the lower limb alignment and excessive stress in the contralateral compartment.

There is currently no unified standard for tibial plateau osteotomy of the UKA, and the optimal PTS should help surgeons perform the proximal tibia osteotomy properly on the sagittal plane, to optimize limb alignment and implant positioning. Many researchers tried different posterior slopes for the tibial plateau osteotomy. Nunley et al. [21] evaluated CT imaging parameters of 2395 patients who had undergone UKA and suggested that to obtain a better knee motor function, the PTS should be within 5° ~ 7°. The degree of PTS greatly influences the kinematics of the knee and plays an important role in sagittal plane stability and tibial translation with weight bearing [23–25]. Weber et al. [8] positioned prostheses under four different tibial slopes (− 4°, 0°, 4°, 8°) to perform a kinematic analysis and found that increasing the tibial slope led to a reduced backside wear. They recommended a tibial slope between 4° and 8° in UKA to reduce wear. Campbell et al. [26] used computer-assisted tomography to perform UKA on 60 patients, whose tibial slopes were at about 7°, and accomplished good postoperative function. Hernigou et al. [27], on the basis of a follow-up of sixteen years (mean time), suggested that the posterior slope of the tibial implant > 7° should be avoided, particularly if the anterior cruciate ligament is absent at the time of implantation. Most importantly, it is generally believed that the anteversion of tibial plateau or a 0° of PTS is undesirable [28, 29], which will not only limit flexion of the knee, but also lead to excessive stress on the tibial prosthesis. It can accelerate the wear of the polyethylene insert and the loosening of the prosthesis. With regard to the re-operation after UKA in our cohort (Table 3), our evidence did not suffice to support a conclusion that PTS is correlated with such failures, and further studies are warranted. However, in this study, we divided the PTS into three groups (< 4°, 4° ~ 7°, and > 7°) and found that too small a PTS (< 4°) led to limited postoperative knee flexion.

**Table 3:** The baseline characteristics and re-operation reasons of the patients

Follow-up patients	Age (y)	BMI (kg·m^− 2^)	PTS (°)	Re-operation	Reasons
Patient 1	73	26.03	3.4	TKA	Infection
Patient 2	66	26.04	9.9	TKA	Unexplained persistent pain
Patient 3	64	29.67	8.8	TKA	Unexplained persistent pain
Patient 4	67	26.37	3.9	TKA	Instability
Patient 5	70	20.62	12.9	Insert exchange	Impingement of residuary cement

Abbreviations: BMI: body mass index, PTS: posterior tibial slope, TKA: total knee arthroplasty

This study has several limitations. First, although studies regarding PTS in UKA are scanty, the retrospective study design is still of limited value. This specific field should be prospectively studied. Second, we did not compare the effects of preoperative and postoperative PTS changes on postoperative knee function, because we believed that the measurement of PTS in the cases of medial compartment knee osteoarthritis was not accurate. Third, the power of this study, like all other radiographic studies of TKA, might be impaired by inter- and intra-observer variability of radiographic measurements. Finally, the size of patients was limited due to the retrospective nature of the study, further larger-sized studies are needed.

In conclusion, although the medial UKA has been successful in patients with medial compartment knee osteoarthritis, the PTS-related knee flexion limitation should not be neglected. It remains a pivotal part of joint replacement surgeries to properly deal with the PTS, since PTS plays a significant role in the maintenance of the tension of cruciate ligaments of the knee joint, the normal sliding and rolling of the femoral condyle, and the stability of the prosthesis. In the tibial plateau osteotomy and fixation of the tibial prosthesis, a too small PTS should be avoided. PTS within an appropriate range (4°∼7°) can help achieve the maximal knee flexion after medial UKA.

## Data Availability

The datasets used and/or analyzed during the current study are available from the corresponding author on reasonable request.

## Abbreviations

PTS: posterior tibial slope
ROM: range of motion
UKA: medial unicompartmental knee arthroplasty
HSS: Hospital for Special Surgery
AP: anteroposterior
BMI: body mass index
TKA: total knee arthroplasty
